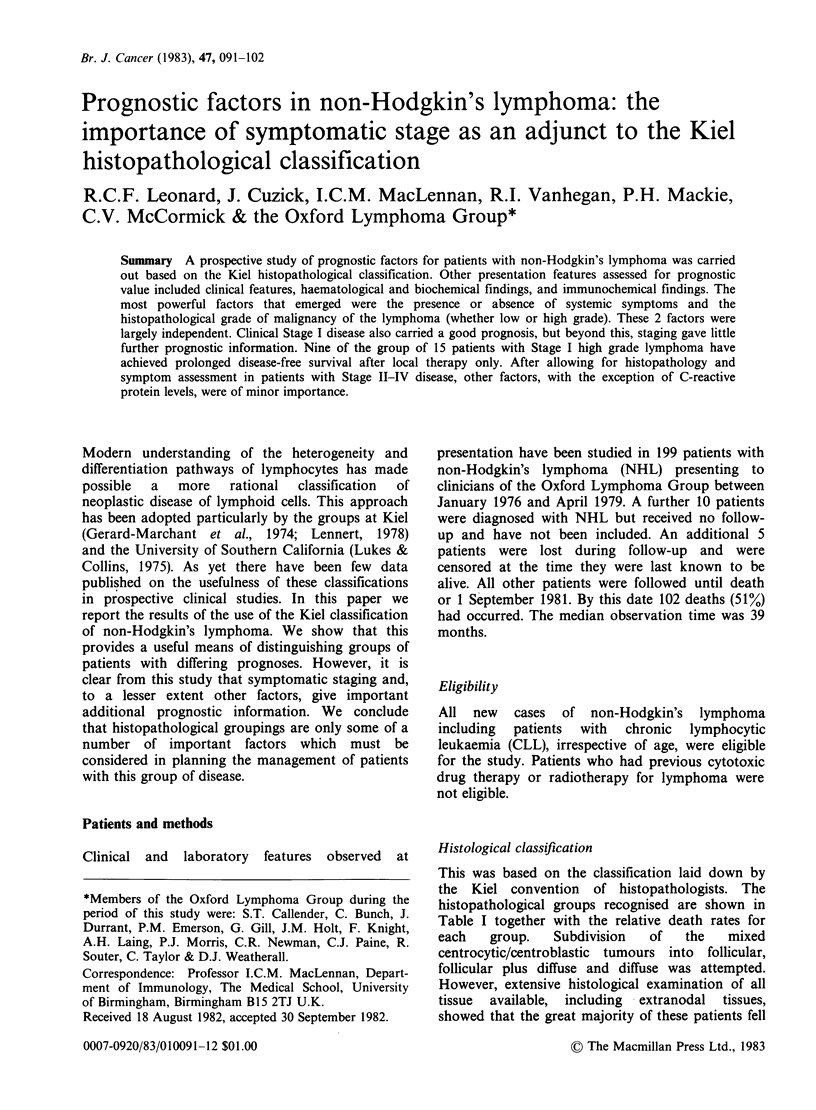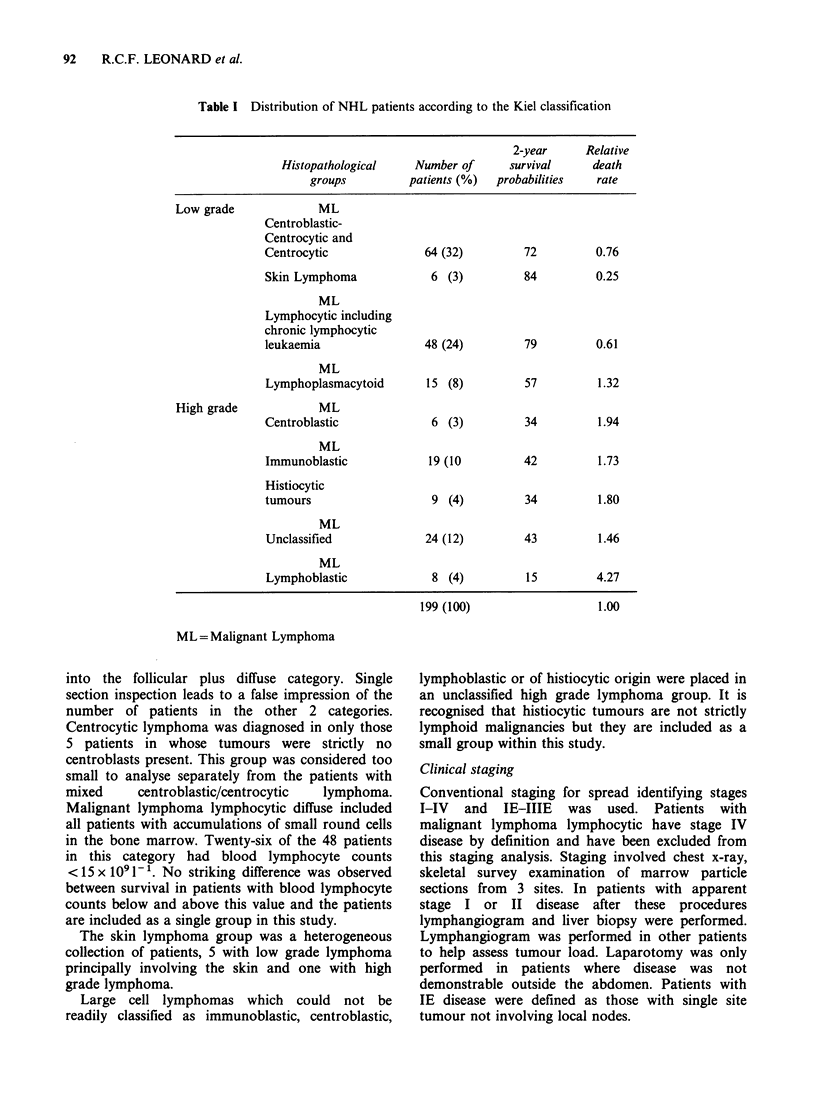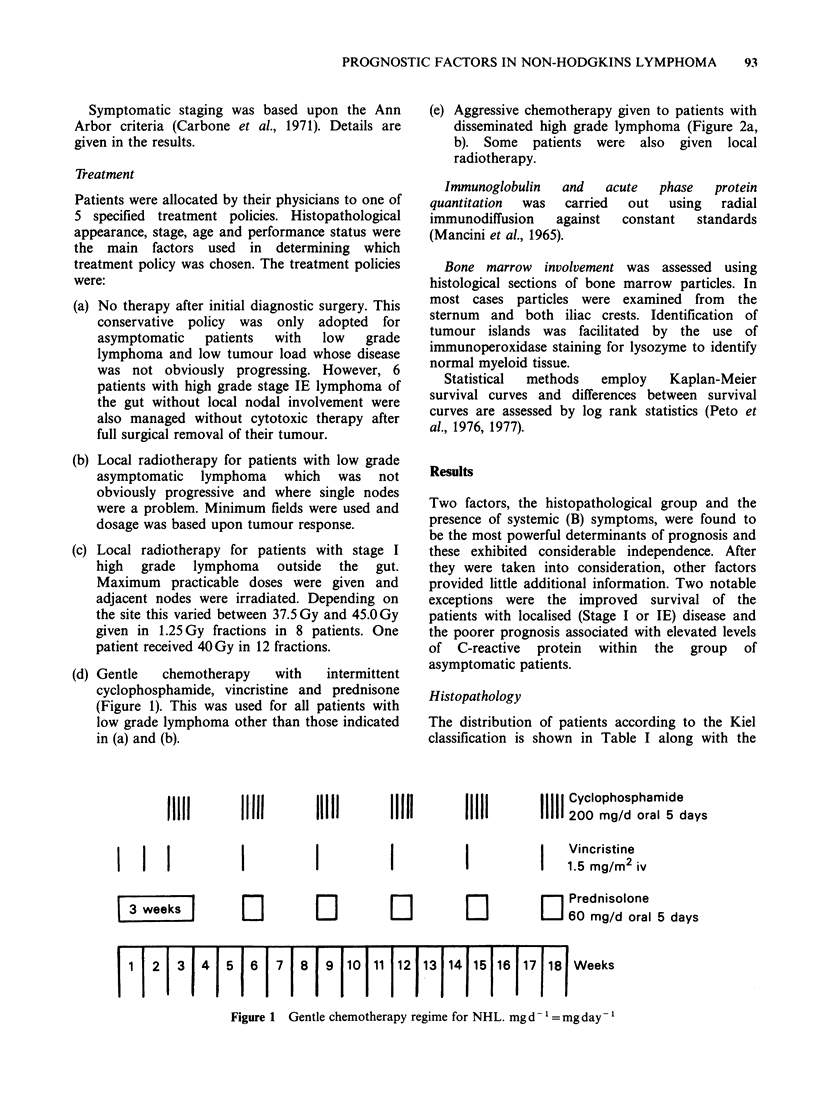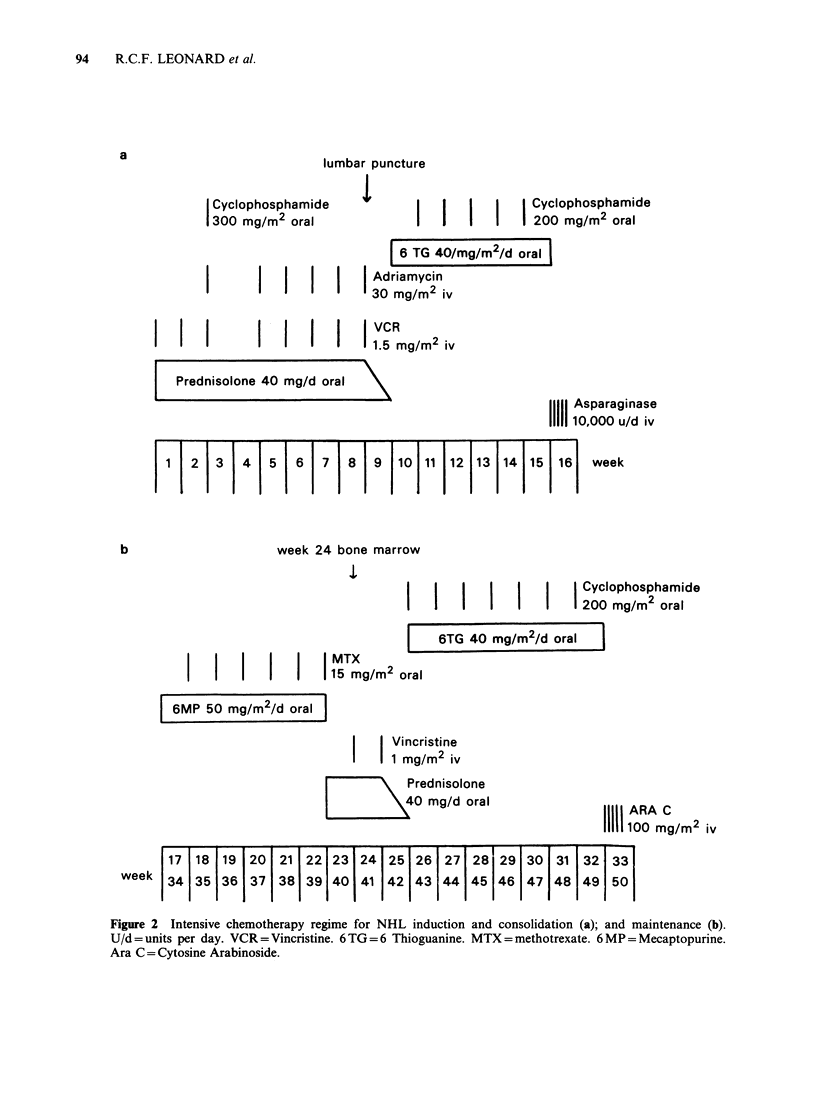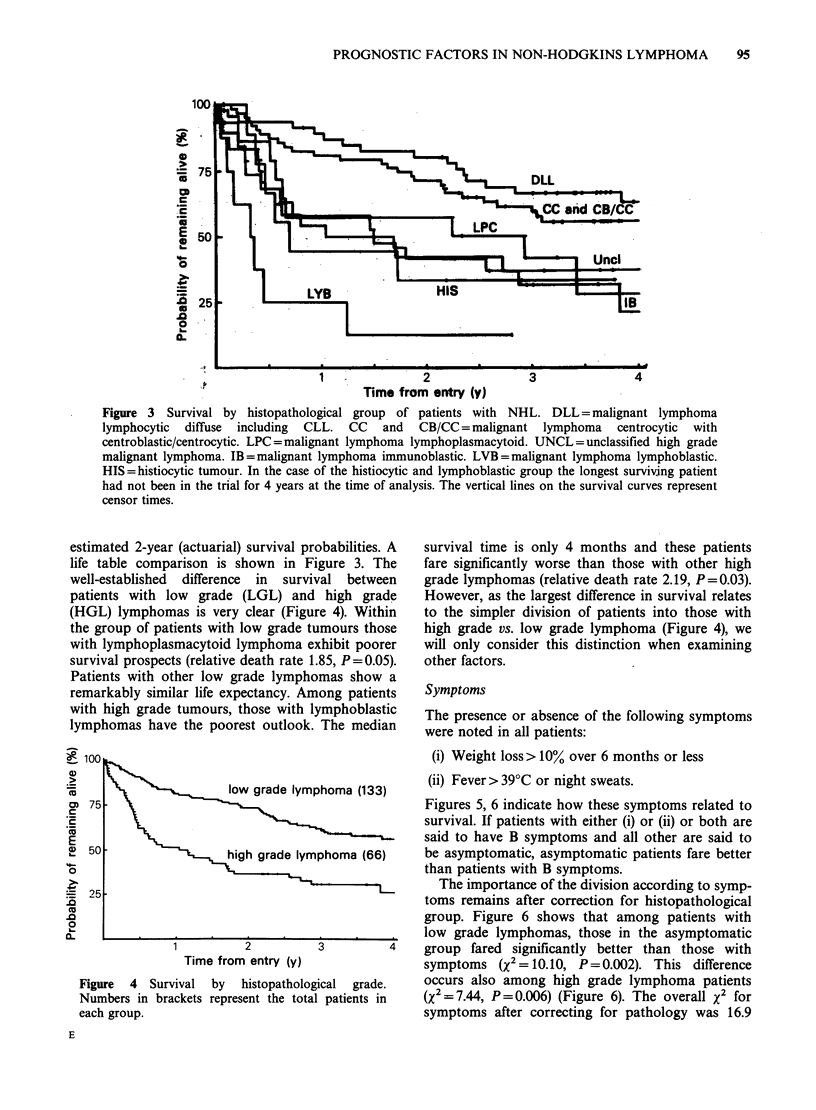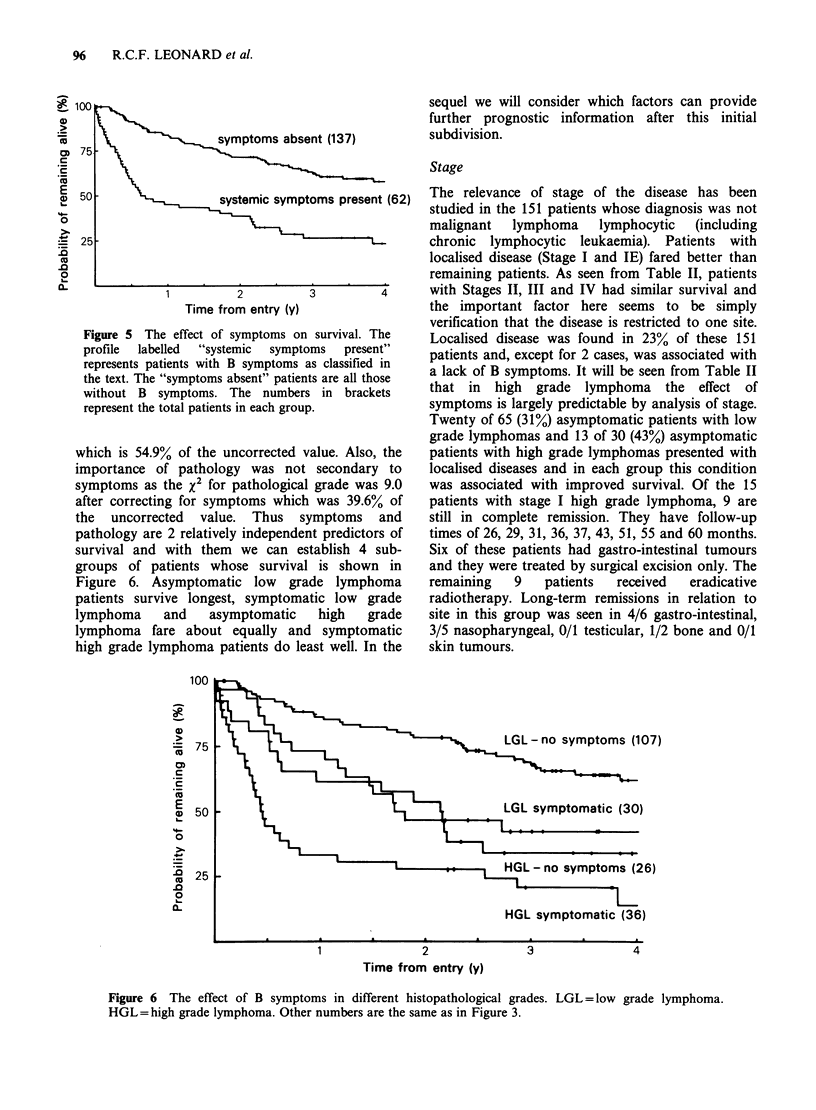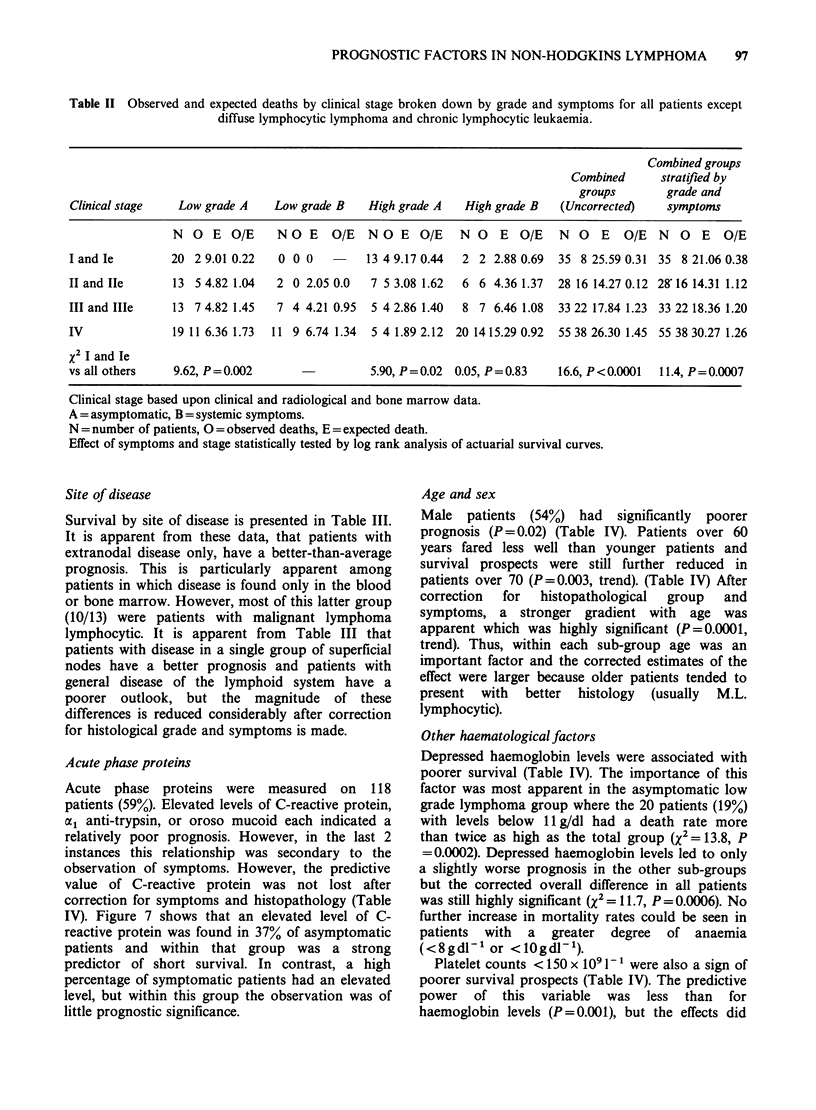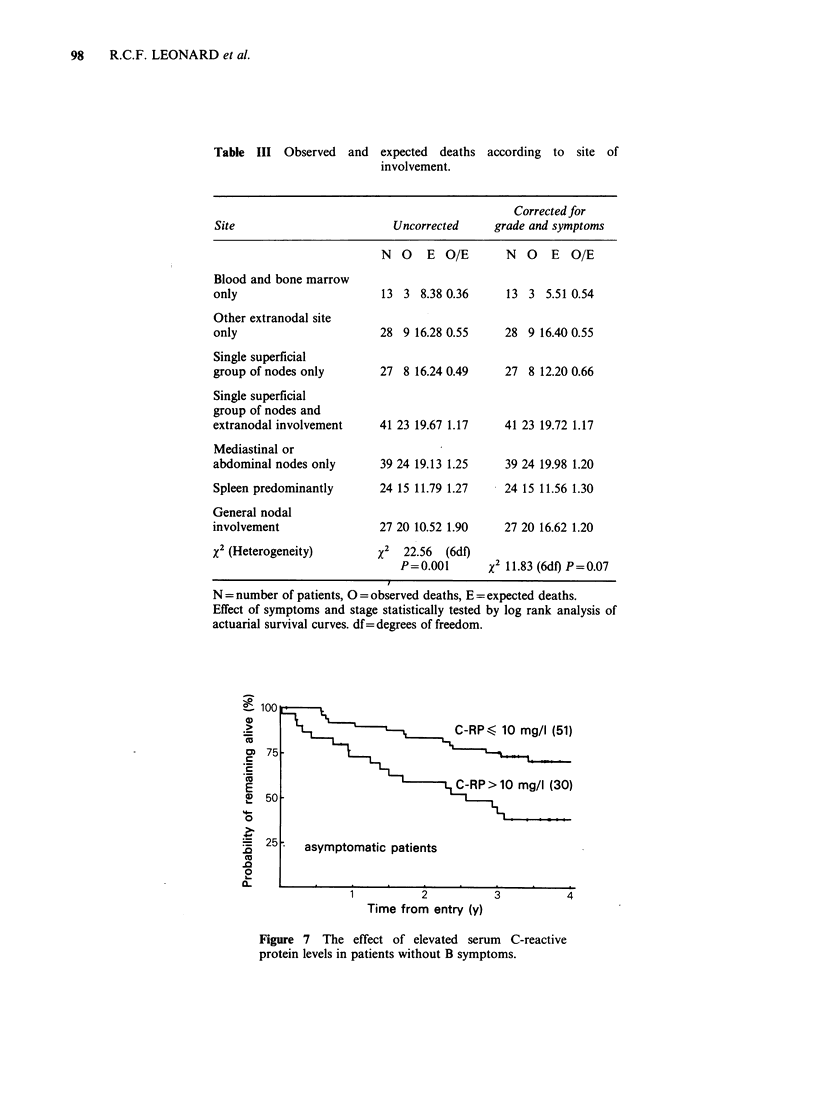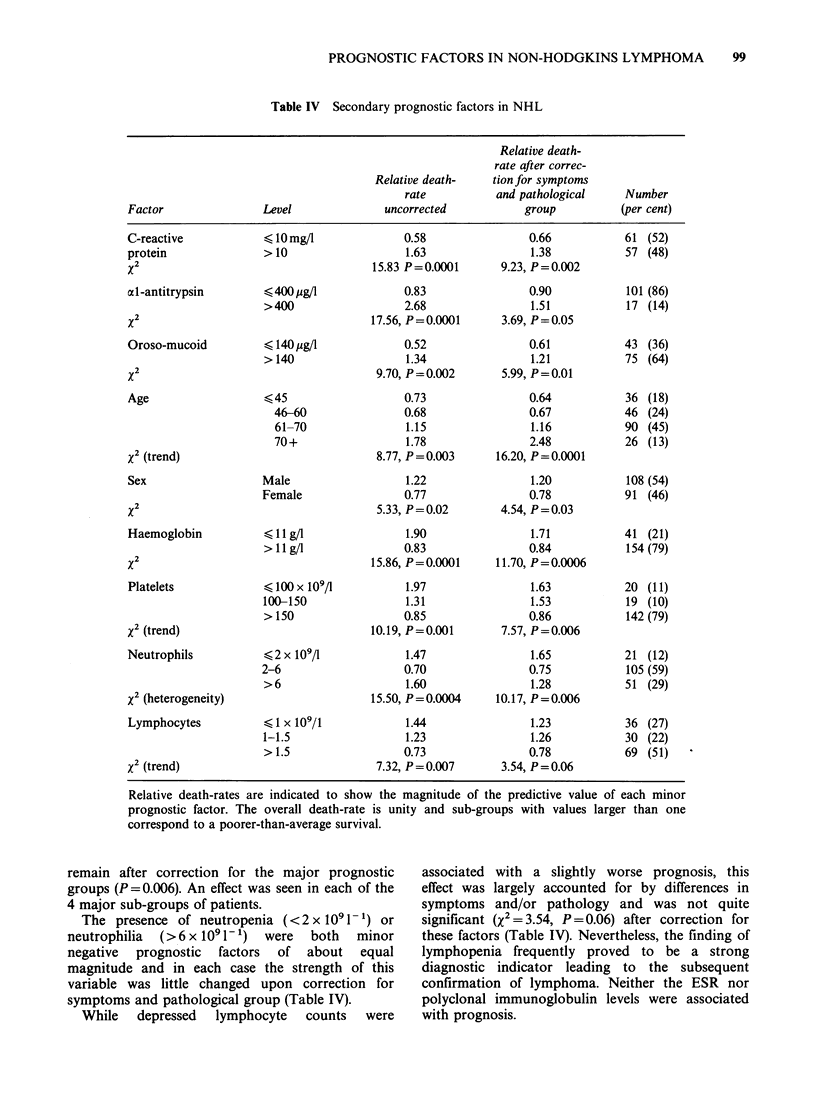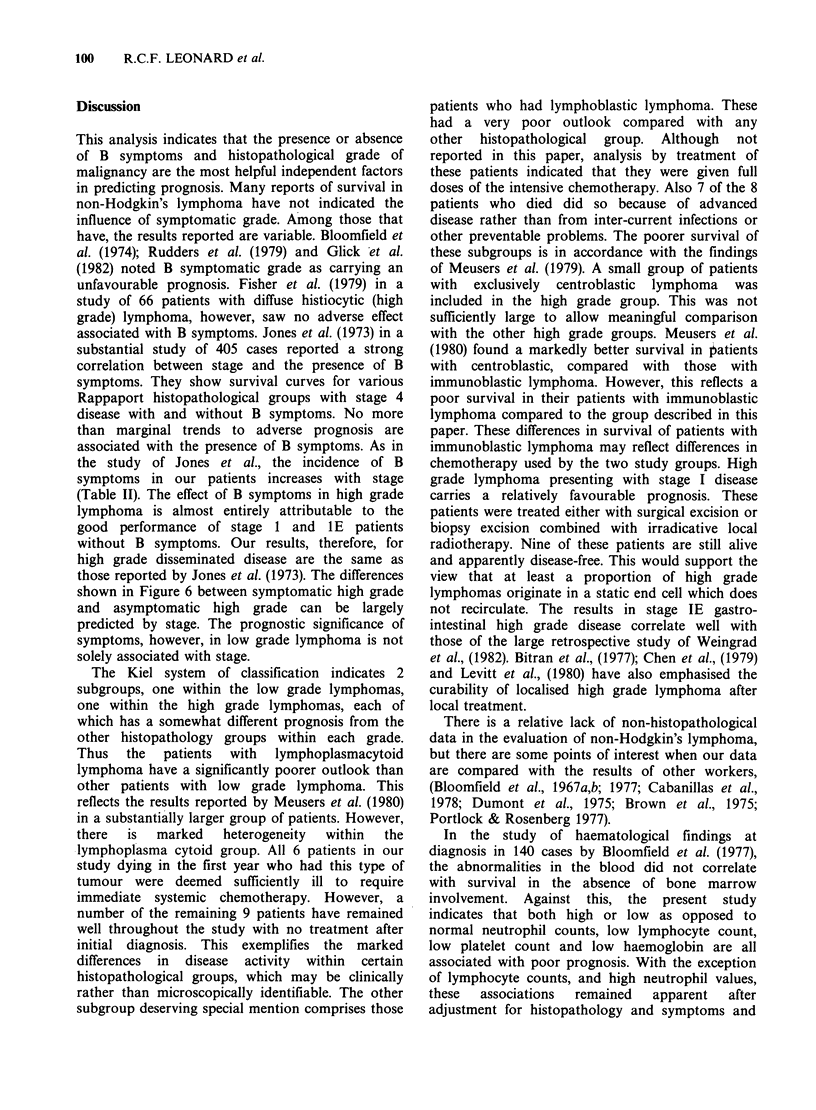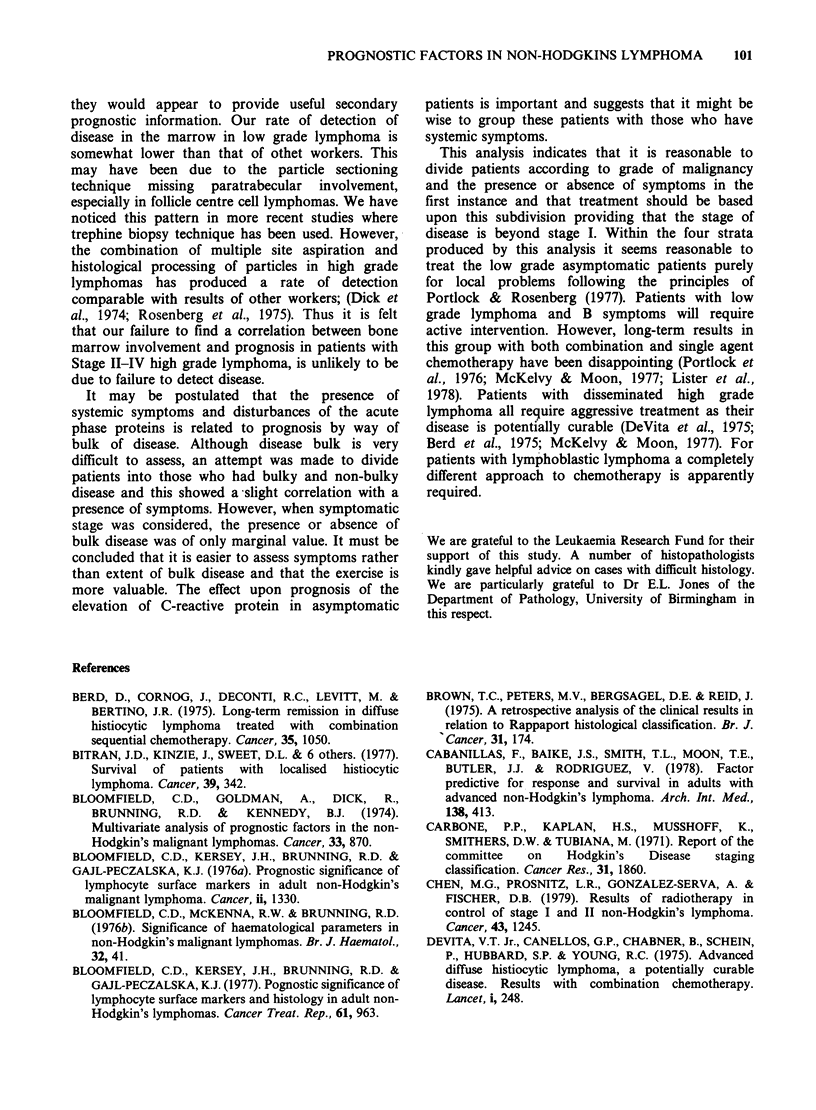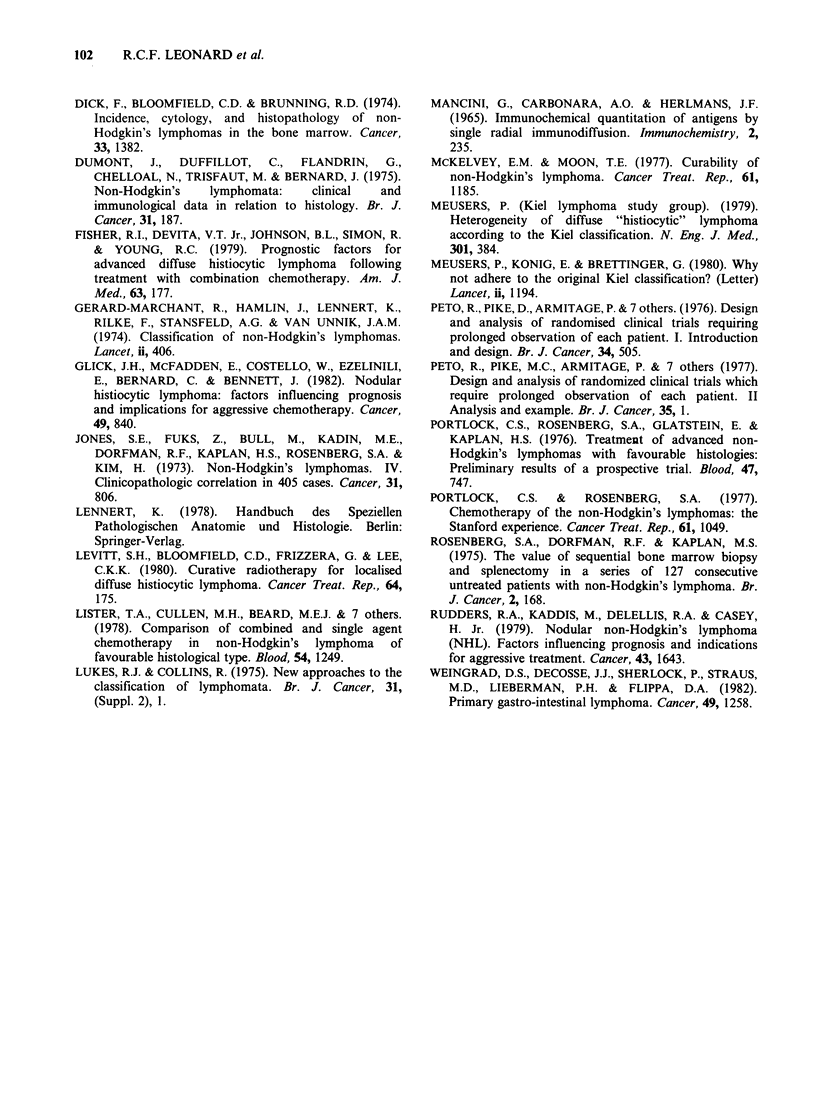# Prognostic factors in non-Hodgkin's lymphoma: the importance of symptomatic stage as an adjunct to the Kiel histopathological classification.

**DOI:** 10.1038/bjc.1983.11

**Published:** 1983-01

**Authors:** R. C. Leonard, J. Cuzick, I. C. MacLennan, R. I. Vanhegan, P. H. Mackie, C. V. McCormick

## Abstract

A prospective study of prognostic factors for patients with non-Hodgkin's lymphoma was carried out based on the Kiel histopathological classification. Other presentation features assessed for prognostic value included clinical features, haematological and biochemical findings, and immunochemical findings. The most powerful factors that emerged were the presence or absence of systemic symptoms and the histopathological grade of malignancy of the lymphoma (whether low or high grade). These 2 factors were largely independent. Clinical Stage I disease also carried a good prognosis, but beyond this, staging gave little further prognostic information. Nine of the group of 15 patients with Stage I high grade lymphoma have achieved prolonged disease-free survival after local therapy only. After allowing for histopathology and symptom assessment in patients with Stage II-IV disease, other factors, with the exception of C-reactive protein levels, were of minor importance.


*Br. J. Cancer* (1983), **47**, 091–102

### Prognostic factors in non-Hodgkin's lymphoma: the importance of symptomatic stage as an adjunct to the Kiel histopathological classification

R.C.F. Leonard, J. Cuzick, I.C.M. MacLennan, R.I. Vanhegan, P.H. Mackie, C.V. McCormick & the Oxford Lymphoma Group*

**Summary** A prospective study of prognostic factors for patients with non-Hodgkin's lymphoma was carried out based on the Kiel histopathological classification. Other presentation features assessed for prognostic value included clinical features, haematological and biochemical findings, and immunochemical findings. The most powerful factors that emerged were the presence or absence of systemic symptoms and the histopathological grade of malignancy of the lymphoma (whether low or high grade). These 2 factors were largely independent. Clinical Stage I disease also carried a good prognosis, but beyond this, staging gave little further prognostic information. Nine of the group of 15 patients with Stage I high grade lymphoma have achieved prolonged disease-free survival after local therapy only. After allowing for histopathology and symptom assessment in patients with Stage II–IV disease, other factors, with the exception of C-reactive protein levels, were of minor importance.

Modern understanding of the heterogeneity and differentiation pathways of lymphocytes has made possible a more rational classification of neoplastic disease of lymphoid cells. This approach has been adopted particularly by the groups at Kiel (Gerard-Marchant *et al.*, 1974; Lennert, 1978) and the University of Southern California (Lukes & Collins, 1975). As yet there have been few data published on the usefulness of these classifications in prospective clinical studies. In this paper we report the results of the use of the Kiel classification of non-Hodgkin's lymphoma. We show that this provides a useful means of distinguishing groups of patients with differing prognoses. However, it is clear from this study that symptomatic staging and, to a lesser extent other factors, give important additional prognostic information. We conclude that histopathological groupings are only some of a number of important factors which must be considered in planning the management of patients with this group of disease.

### Patients and methods

Clinical and laboratory features observed at presentation have been studied in 199 patients with non-Hodgkin's lymphoma (NHL) presenting to clinicians of the Oxford Lymphoma Group between January 1976 and April 1979. A further 10 patients were diagnosed with NHL but received no follow-up and have not been included. An additional 5 patients were lost during follow-up and were censored at the time they were last known to be alive. All other patients were followed until death or 1 September 1981. By this date 102 deaths (51%) had occurred. The median observation time was 39 months.

### *Eligibility*

All new cases of non-Hodgkin's lymphoma including patients with chronic lymphocytic leukaemia (CLL), irrespective of age, were eligible for the study. Patients who had previous cytotoxic drug therapy or radiotherapy for lymphoma were not eligible.

### *Histological classification*

This was based on the classification laid down by the Kiel convention of histopathologists. The histopathological groups recognised are shown in Table I together with the relative death rates for each group. Subdivision of the mixed centrocytic/centroblastic tumours into follicular, follicular plus diffuse and diffuse was attempted. However, extensive histological examination of all tissue available, including extranodal tissues, showed that the great majority of these patients fell

*Members of the Oxford Lymphoma Group during the period of this study were: S.T. Callender, C. Bunch, J. Durrant, P.M. Emerson, G. Gill, J.M. Holt, F. Knight, A.H. Laing, P.J. Morris, C.R. Newman, C.J. Paine, R. Souter, C. Taylor & D.J. Weatherall.

Correspondence: Professor I.C.M. MacLennan, Department of Immunology, The Medical School, University of Birmingham, Birmingham B15 2TJ U.K.

Received 18 August 1982, accepted 30 September 1982.

0007-0920/83/010091-12 $01.00 © The Macmillan Press Ltd., 1983

**Table I** Distribution of NHL patients according to the Kiel classification

| | *Histopathological groups* | *Number of patients* (%) | *2-year survival probabilities* | *Relative death rate* |
|---|---|---|---|---|
| Low grade | ML Centroblastic-Centrocytic and Centrocytic | 64 (32) | 72 | 0.76 |
| | Skin Lymphoma | 6 (3) | 84 | 0.25 |
| | ML Lymphocytic including chronic lymphocytic leukaemia | 48 (24) | 79 | 0.61 |
| | ML Lymphoplasmacytoid | 15 (8) | 57 | 1.32 |
| High grade | ML Centroblastic | 6 (3) | 34 | 1.94 |
| | ML Immunoblastic | 19 (10 | 42 | 1.73 |
| | Histiocytic tumours | 9 (4) | 34 | 1.80 |
| | ML Unclassified | 24 (12) | 43 | 1.46 |
| | ML Lymphoblastic | 8 (4) | 15 | 4.27 |
| | | 199 (100) | | 1.00 |

ML = Malignant Lymphoma into the follicular plus diffuse category. Single section inspection leads to a false impression of the number of patients in the other 2 categories. Centrocytic lymphoma was diagnosed in only those 5 patients in whose tumours were strictly no centroblasts present. This group was considered too small to analyse separately from the patients with mixed centroblastic/centrocytic lymphoma. Malignant lymphoma lymphocytic diffuse included all patients with accumulations of small round cells in the bone marrow. Twenty-six of the 48 patients in this category had blood lymphocyte counts $<15\times10^{9}\,l^{-1}$. No striking difference was observed between survival in patients with blood lymphocyte counts below and above this value and the patients are included as a single group in this study.

The skin lymphoma group was a heterogeneous collection of patients, 5 with low grade lymphoma principally involving the skin and one with high grade lymphoma.

Large cell lymphomas which could not be readily classified as immunoblastic, centroblastic, lymphoblastic or of histiocytic origin were placed in an unclassified high grade lymphoma group. It is recognised that histiocytic tumours are not strictly lymphoid malignancies but they are included as a small group within this study.

*Clinical staging*

Conventional staging for spread identifying stages I–IV and IE–IIIE was used. Patients with malignant lymphoma lymphocytic have stage IV disease by definition and have been excluded from this staging analysis. Staging involved chest x-ray, skeletal survey examination of marrow particle sections from 3 sites. In patients with apparent stage I or II disease after these procedures lymphangiogram and liver biopsy were performed. Lymphangiogram was performed in other patients to help assess tumour load. Laparotomy was only performed in patients where disease was not demonstrable outside the abdomen. Patients with IE disease were defined as those with single site tumour not involving local nodes.

Symptomatic staging was based upon the Ann Arbor criteria (Carbone *et al.*, 1971). Details are given in the results.

*Treatment*

Patients were allocated by their physicians to one of 5 specified treatment policies. Histopathological appearance, stage, age and performance status were the main factors used in determining which treatment policy was chosen. The treatment policies were:

(a) No therapy after initial diagnostic surgery. This conservative policy was only adopted for asymptomatic patients with low grade lymphoma and low tumour load whose disease was not obviously progressing. However, 6 patients with high grade stage IE lymphoma of the gut without local nodal involvement were also managed without cytotoxic therapy after full surgical removal of their tumour.

(b) Local radiotherapy for patients with low grade asymptomatic lymphoma which was not obviously progressive and where single nodes were a problem. Minimum fields were used and dosage was based upon tumour response.

(c) Local radiotherapy for patients with stage I high grade lymphoma outside the gut. Maximum practicable doses were given and adjacent nodes were irradiated. Depending on the site this varied between 37.5 Gy and 45.0 Gy given in 1.25 Gy fractions in 8 patients. One patient received 40 Gy in 12 fractions.

(d) Gentle chemotherapy with intermittent cyclophosphamide, vincristine and prednisone (Figure 1). This was used for all patients with low grade lymphoma other than those indicated in (a) and (b).

(e) Aggressive chemotherapy given to patients with disseminated high grade lymphoma (Figure 2a, b). Some patients were also given local radiotherapy.

*Immunoglobulin and acute phase protein quantitation* was carried out using radial immunodiffusion against constant standards (Mancini *et al.*, 1965).

*Bone marrow involvement* was assessed using histological sections of bone marrow particles. In most cases particles were examined from the sternum and both iliac crests. Identification of tumour islands was facilitated by the use of immunoperoxidase staining for lysozyme to identify normal myeloid tissue.

Statistical methods employ Kaplan-Meier survival curves and differences between survival curves are assessed by log rank statistics (Peto *et al.*, 1976, 1977).

**Results**

Two factors, the histopathological group and the presence of systemic (B) symptoms, were found to be the most powerful determinants of prognosis and these exhibited considerable independence. After they were taken into consideration, other factors provided little additional information. Two notable exceptions were the improved survival of the patients with localised (Stage I or IE) disease and the poorer prognosis associated with elevated levels of C-reactive protein within the group of asymptomatic patients.

*Histopathology*

The distribution of patients according to the Kiel classification is shown in Table I along with the

**Figure 1** Gentle chemotherapy regime for NHL. $mg\,d^{-1} = mg\,day^{-1}$

**Figure 2** Intensive chemotherapy regime for NHL induction and consolidation (**a**); and maintenance (**b**). U/d = units per day. VCR = Vincristine. 6TG = 6 Thioguanine. MTX = methotrexate. 6MP = Mecaptopurine. Ara C = Cytosine Arabinoside.

**Figure 3** Survival by histopathological group of patients with NHL. DLL = malignant lymphoma lymphocytic diffuse including CLL. CC and CB/CC = malignant lymphoma centrocytic with centroblastic/centrocytic. LPC = malignant lymphoma lymphoplasmacytoid. UNCL = unclassified high grade malignant lymphoma. IB = malignant lymphoma immunoblastic. LVB = malignant lymphoma lymphoblastic. HIS = histiocytic tumour. In the case of the histiocytic and lymphoblastic group the longest surviving patient had not been in the trial for 4 years at the time of analysis. The vertical lines on the survival curves represent censor times.

estimated 2-year (actuarial) survival probabilities. A life table comparison is shown in Figure 3. The well-established difference in survival between patients with low grade (LGL) and high grade (HGL) lymphomas is very clear (Figure 4). Within the group of patients with low grade tumours those with lymphoplasmacytoid lymphoma exhibit poorer survival prospects (relative death rate 1.85, $P = 0.05$). Patients with other low grade lymphomas show a remarkably similar life expectancy. Among patients with high grade tumours, those with lymphoblastic lymphomas have the poorest outlook. The median

**Figure 4** Survival by histopathological grade. Numbers in brackets represent the total patients in each group.

survival time is only 4 months and these patients fare significantly worse than those with other high grade lymphomas (relative death rate 2.19, $P = 0.03$). However, as the largest difference in survival relates to the simpler division of patients into those with high grade *vs.* low grade lymphoma (Figure 4), we will only consider this distinction when examining other factors.

*Symptoms*

The presence or absence of the following symptoms were noted in all patients:

(i) Weight loss > 10% over 6 months or less

(ii) Fever > 39°C or night sweats.

Figures 5, 6 indicate how these symptoms related to survival. If patients with either (i) or (ii) or both are said to have B symptoms and all other are said to be asymptomatic, asymptomatic patients fare better than patients with B symptoms.

The importance of the division according to symptoms remains after correction for histopathological group. Figure 6 shows that among patients with low grade lymphomas, those in the asymptomatic group fared significantly better than those with symptoms ($\chi^2 = 10.10$, $P = 0.002$). This difference occurs also among high grade lymphoma patients ($\chi^2 = 7.44$, $P = 0.006$) (Figure 6). The overall $\chi^2$ for symptoms after correcting for pathology was 16.9

**Figure 5** The effect of symptoms on survival. The profile labelled "systemic symptoms present" represents patients with B symptoms as classified in the text. The "symptoms absent" patients are all those without B symptoms. The numbers in brackets represent the total patients in each group.

which is 54.9% of the uncorrected value. Also, the importance of pathology was not secondary to symptoms as the $\chi^2$ for pathological grade was 9.0 after correcting for symptoms which was 39.6% of the uncorrected value. Thus symptoms and pathology are 2 relatively independent predictors of survival and with them we can establish 4 subgroups of patients whose survival is shown in Figure 6. Asymptomatic low grade lymphoma patients survive longest, symptomatic low grade lymphoma and asymptomatic high grade lymphoma fare about equally and symptomatic high grade lymphoma patients do least well. In the sequel we will consider which factors can provide further prognostic information after this initial subdivision.

*Stage*

The relevance of stage of the disease has been studied in the 151 patients whose diagnosis was not malignant lymphoma lymphocytic (including chronic lymphocytic leukaemia). Patients with localised disease (Stage I and IE) fared better than remaining patients. As seen from Table II, patients with Stages II, III and IV had similar survival and the important factor here seems to be simply verification that the disease is restricted to one site. Localised disease was found in 23% of these 151 patients and, except for 2 cases, was associated with a lack of B symptoms. It will be seen from Table II that in high grade lymphoma the effect of symptoms is largely predictable by analysis of stage. Twenty of 65 (31%) asymptomatic patients with low grade lymphomas and 13 of 30 (43%) asymptomatic patients with high grade lymphomas presented with localised diseases and in each group this condition was associated with improved survival. Of the 15 patients with stage I high grade lymphoma, 9 are still in complete remission. They have follow-up times of 26, 29, 31, 36, 37, 43, 51, 55 and 60 months. Six of these patients had gastro-intestinal tumours and they were treated by surgical excision only. The remaining 9 patients received eradicative radiotherapy. Long-term remissions in relation to site in this group was seen in 4/6 gastro-intestinal, 3/5 nasopharyngeal, 0/1 testicular, 1/2 bone and 0/1 skin tumours.

**Figure 6** The effect of B symptoms in different histopathological grades. LGL = low grade lymphoma. HGL = high grade lymphoma. Other numbers are the same as in Figure 3.

**Table II** Observed and expected deaths by clinical stage broken down by grade and symptoms for all patients except diffuse lymphocytic lymphoma and chronic lymphocytic leukaemia.

| *Clinical stage* | *Low grade A* | | | | *Low grade B* | | | | *High grade A* | | | | *High grade B* | | | | *Combined groups (Uncorrected)* | | | | *Combined groups stratified by grade and symptoms* | | | |
|---|---|---|---|---|---|---|---|---|---|---|---|---|---|---|---|---|---|---|---|---|---|---|---|---|
| | N | O | E | O/E | N | O | E | O/E | N | O | E | O/E | N | O | E | O/E | N | O | E | O/E | N | O | E | O/E |
| I and Ie | 20 | 2 | 9.01 | 0.22 | 0 | 0 | 0 | — | 13 | 4 | 9.17 | 0.44 | 2 | 2 | 2.88 | 0.69 | 35 | 8 | 25.59 | 0.31 | 35 | 8 | 21.06 | 0.38 |
| II and IIe | 13 | 5 | 4.82 | 1.04 | 2 | 0 | 2.05 | 0.0 | 7 | 5 | 3.08 | 1.62 | 6 | 6 | 4.36 | 1.37 | 28 | 16 | 14.27 | 0.12 | 28 | 16 | 14.31 | 1.12 |
| III and IIIe | 13 | 7 | 4.82 | 1.45 | 7 | 4 | 4.21 | 0.95 | 5 | 4 | 2.86 | 1.40 | 8 | 7 | 6.46 | 1.08 | 33 | 22 | 17.84 | 1.23 | 33 | 22 | 18.36 | 1.20 |
| IV | 19 | 11 | 6.36 | 1.73 | 11 | 9 | 6.74 | 1.34 | 5 | 4 | 1.89 | 2.12 | 20 | 14 | 15.29 | 0.92 | 55 | 38 | 26.30 | 1.45 | 55 | 38 | 30.27 | 1.26 |
| $\chi^2$ I and Ie vs all others | 9.62, $P=0.002$ | | | | — | | | | 5.90, $P=0.02$ | | | | 0.05, $P=0.83$ | | | | 16.6, $P<0.0001$ | | | | 11.4, $P=0.0007$ | | | |

Clinical stage based upon clinical and radiological and bone marrow data.
A = asymptomatic, B = systemic symptoms.
N = number of patients, O = observed deaths, E = expected death.
Effect of symptoms and stage statistically tested by log rank analysis of actuarial survival curves.

*Site of disease*

Survival by site of disease is presented in Table III. It is apparent from these data, that patients with extranodal disease only, have a better-than-average prognosis. This is particularly apparent among patients in which disease is found only in the blood or bone marrow. However, most of this latter group (10/13) were patients with malignant lymphoma lymphocytic. It is apparent from Table III that patients with disease in a single group of superficial nodes have a better prognosis and patients with general disease of the lymphoid system have a poorer outlook, but the magnitude of these differences is reduced considerably after correction for histological grade and symptoms is made.

*Acute phase proteins*

Acute phase proteins were measured on 118 patients (59%). Elevated levels of C-reactive protein, $\alpha_1$ anti-trypsin, or oroso mucoid each indicated a relatively poor prognosis. However, in the last 2 instances this relationship was secondary to the observation of symptoms. However, the predictive value of C-reactive protein was not lost after correction for symptoms and histopathology (Table IV). Figure 7 shows that an elevated level of C-reactive protein was found in 37% of asymptomatic patients and within that group was a strong predictor of short survival. In contrast, a high percentage of symptomatic patients had an elevated level, but within this group the observation was of little prognostic significance.

*Age and sex*

Male patients (54%) had significantly poorer prognosis ($P=0.02$) (Table IV). Patients over 60 years fared less well than younger patients and survival prospects were still further reduced in patients over 70 ($P=0.003$, trend). (Table IV) After correction for histopathological group and symptoms, a stronger gradient with age was apparent which was highly significant ($P=0.0001$, trend). Thus, within each sub-group age was an important factor and the corrected estimates of the effect were larger because older patients tended to present with better histology (usually M.L. lymphocytic).

*Other haematological factors*

Depressed haemoglobin levels were associated with poorer survival (Table IV). The importance of this factor was most apparent in the asymptomatic low grade lymphoma group where the 20 patients (19%) with levels below 11 g/dl had a death rate more than twice as high as the total group ($\chi^2=13.8$, $P=0.0002$). Depressed haemoglobin levels led to only a slightly worse prognosis in the other sub-groups but the corrected overall difference in all patients was still highly significant ($\chi^2=11.7$, $P=0.0006$). No further increase in mortality rates could be seen in patients with a greater degree of anaemia ($<8\,g\,dl^{-1}$ or $<10\,g\,dl^{-1}$).

Platelet counts $<150\times10^9\,l^{-1}$ were also a sign of poorer survival prospects (Table IV). The predictive power of this variable was less than for haemoglobin levels ($P=0.001$), but the effects did

**Table III** Observed and expected deaths according to site of involvement.

| *Site* | *Uncorrected* | | | | *Corrected for grade and symptoms* | | | |
|---|---|---|---|---|---|---|---|---|
| | N | O | E | O/E | N | O | E | O/E |
| Blood and bone marrow only | 13 | 3 | 8.38 | 0.36 | 13 | 3 | 5.51 | 0.54 |
| Other extranodal site only | 28 | 9 | 16.28 | 0.55 | 28 | 9 | 16.40 | 0.55 |
| Single superficial group of nodes only | 27 | 8 | 16.24 | 0.49 | 27 | 8 | 12.20 | 0.66 |
| Single superficial group of nodes and extranodal involvement | 41 | 23 | 19.67 | 1.17 | 41 | 23 | 19.72 | 1.17 |
| Mediastinal or abdominal nodes only | 39 | 24 | 19.13 | 1.25 | 39 | 24 | 19.98 | 1.20 |
| Spleen predominantly | 24 | 15 | 11.79 | 1.27 | 24 | 15 | 11.56 | 1.30 |
| General nodal involvement | 27 | 20 | 10.52 | 1.90 | 27 | 20 | 16.62 | 1.20 |
| $\chi^2$ (Heterogeneity) | $\chi^2$ 22.56 (6df) $P=0.001$ | | | | $\chi^2$ 11.83 (6df) $P=0.07$ | | | |

N = number of patients, O = observed deaths, E = expected deaths.
Effect of symptoms and stage statistically tested by log rank analysis of actuarial survival curves. df = degrees of freedom.

**Figure 7** The effect of elevated serum C-reactive protein levels in patients without B symptoms.

**Table IV** Secondary prognostic factors in NHL

| *Factor* | *Level* | *Relative death-rate uncorrected* | *Relative death-rate after correction for symptoms and pathological group* | *Number (per cent)* |
|---|---|---|---|---|
| C-reactive | $\leqslant$10 mg/l | 0.58 | 0.66 | 61 (52) |
| protein | >10 | 1.63 | 1.38 | 57 (48) |
| $\chi^2$ | | 15.83 $P$=0.0001 | 9.23, $P$=0.002 | |
| α1-antitrypsin | $\leqslant$400 μg/l | 0.83 | 0.90 | 101 (86) |
| | >400 | 2.68 | 1.51 | 17 (14) |
| $\chi^2$ | | 17.56, $P$=0.0001 | 3.69, $P$=0.05 | |
| Oroso-mucoid | $\leqslant$140 μg/l | 0.52 | 0.61 | 43 (36) |
| | >140 | 1.34 | 1.21 | 75 (64) |
| $\chi^2$ | | 9.70, $P$=0.002 | 5.99, $P$=0.01 | |
| Age | $\leqslant$45 | 0.73 | 0.64 | 36 (18) |
| | 46–60 | 0.68 | 0.67 | 46 (24) |
| | 61–70 | 1.15 | 1.16 | 90 (45) |
| | 70+ | 1.78 | 2.48 | 26 (13) |
| $\chi^2$ (trend) | | 8.77, $P$=0.003 | 16.20, $P$=0.0001 | |
| Sex | Male | 1.22 | 1.20 | 108 (54) |
| | Female | 0.77 | 0.78 | 91 (46) |
| $\chi^2$ | | 5.33, $P$=0.02 | 4.54, $P$=0.03 | |
| Haemoglobin | $\leqslant$11 g/l | 1.90 | 1.71 | 41 (21) |
| | >11 g/l | 0.83 | 0.84 | 154 (79) |
| $\chi^2$ | | 15.86, $P$=0.0001 | 11.70, $P$=0.0006 | |
| Platelets | $\leqslant 100 \times 10^9$/l | 1.97 | 1.63 | 20 (11) |
| | 100–150 | 1.31 | 1.53 | 19 (10) |
| | >150 | 0.85 | 0.86 | 142 (79) |
| $\chi^2$ (trend) | | 10.19, $P$=0.001 | 7.57, $P$=0.006 | |
| Neutrophils | $\leqslant 2 \times 10^9$/l | 1.47 | 1.65 | 21 (12) |
| | 2–6 | 0.70 | 0.75 | 105 (59) |
| | >6 | 1.60 | 1.28 | 51 (29) |
| $\chi^2$ (heterogeneity) | | 15.50, $P$=0.0004 | 10.17, $P$=0.006 | |
| Lymphocytes | $\leqslant 1 \times 10^9$/1 | 1.44 | 1.23 | 36 (27) |
| | 1–1.5 | 1.23 | 1.26 | 30 (22) |
| | >1.5 | 0.73 | 0.78 | 69 (51) |
| $\chi^2$ (trend) | | 7.32, $P$=0.007 | 3.54, $P$=0.06 | |

Relative death-rates are indicated to show the magnitude of the predictive value of each minor prognostic factor. The overall death-rate is unity and sub-groups with values larger than one correspond to a poorer-than-average survival.

remain after correction for the major prognostic groups ($P$=0.006). An effect was seen in each of the 4 major sub-groups of patients.

The presence of neutropenia ($<2 \times 10^9 l^{-1}$) or neutrophilia ($>6 \times 10^9 l^{-1}$) were both minor negative prognostic factors of about equal magnitude and in each case the strength of this variable was little changed upon correction for symptoms and pathological group (Table IV).

While depressed lymphocyte counts were associated with a slightly worse prognosis, this effect was largely accounted for by differences in symptoms and/or pathology and was not quite significant ($\chi^2$=3.54, $P$=0.06) after correction for these factors (Table IV). Nevertheless, the finding of lymphopenia frequently proved to be a strong diagnostic indicator leading to the subsequent confirmation of lymphoma. Neither the ESR nor polyclonal immunoglobulin levels were associated with prognosis.

### Discussion

This analysis indicates that the presence or absence of B symptoms and histopathological grade of malignancy are the most helpful independent factors in predicting prognosis. Many reports of survival in non-Hodgkin's lymphoma have not indicated the influence of symptomatic grade. Among those that have, the results reported are variable. Bloomfield *et al.* (1974); Rudders *et al.* (1979) and Glick *et al.* (1982) noted B symptomatic grade as carrying an unfavourable prognosis. Fisher *et al.* (1979) in a study of 66 patients with diffuse histiocytic (high grade) lymphoma, however, saw no adverse effect associated with B symptoms. Jones *et al.* (1973) in a substantial study of 405 cases reported a strong correlation between stage and the presence of B symptoms. They show survival curves for various Rappaport histopathological groups with stage 4 disease with and without B symptoms. No more than marginal trends to adverse prognosis are associated with the presence of B symptoms. As in the study of Jones *et al.*, the incidence of B symptoms in our patients increases with stage (Table II). The effect of B symptoms in high grade lymphoma is almost entirely attributable to the good performance of stage 1 and 1E patients without B symptoms. Our results, therefore, for high grade disseminated disease are the same as those reported by Jones *et al.* (1973). The differences shown in Figure 6 between symptomatic high grade and asymptomatic high grade can be largely predicted by stage. The prognostic significance of symptoms, however, in low grade lymphoma is not solely associated with stage.

The Kiel system of classification indicates 2 subgroups, one within the low grade lymphomas, one within the high grade lymphomas, each of which has a somewhat different prognosis from the other histopathology groups within each grade. Thus the patients with lymphoplasmacytoid lymphoma have a significantly poorer outlook than other patients with low grade lymphoma. This reflects the results reported by Meusers *et al.* (1980) in a substantially larger group of patients. However, there is marked heterogeneity within the lymphoplasma cytoid group. All 6 patients in our study dying in the first year who had this type of tumour were deemed sufficiently ill to require immediate systemic chemotherapy. However, a number of the remaining 9 patients have remained well throughout the study with no treatment after initial diagnosis. This exemplifies the marked differences in disease activity within certain histopathological groups, which may be clinically rather than microscopically identifiable. The other subgroup deserving special mention comprises those patients who had lymphoblastic lymphoma. These had a very poor outlook compared with any other histopathological group. Although not reported in this paper, analysis by treatment of these patients indicated that they were given full doses of the intensive chemotherapy. Also 7 of the 8 patients who died did so because of advanced disease rather than from inter-current infections or other preventable problems. The poorer survival of these subgroups is in accordance with the findings of Meusers *et al.* (1979). A small group of patients with exclusively centroblastic lymphoma was included in the high grade group. This was not sufficiently large to allow meaningful comparison with the other high grade groups. Meusers *et al.* (1980) found a markedly better survival in patients with centroblastic, compared with those with immunoblastic lymphoma. However, this reflects a poor survival in their patients with immunoblastic lymphoma compared to the group described in this paper. These differences in survival of patients with immunoblastic lymphoma may reflect differences in chemotherapy used by the two study groups. High grade lymphoma presenting with stage I disease carries a relatively favourable prognosis. These patients were treated either with surgical excision or biopsy excision combined with irradicative local radiotherapy. Nine of these patients are still alive and apparently disease-free. This would support the view that at least a proportion of high grade lymphomas originate in a static end cell which does not recirculate. The results in stage IE gastro-intestinal high grade disease correlate well with those of the large retrospective study of Weingrad *et al.*, (1982). Bitran *et al.*, (1977); Chen *et al.*, (1979) and Levitt *et al.*, (1980) have also emphasised the curability of localised high grade lymphoma after local treatment.

There is a relative lack of non-histopathological data in the evaluation of non-Hodgkin's lymphoma, but there are some points of interest when our data are compared with the results of other workers, (Bloomfield *et al.*, 1967*a,b*; 1977; Cabanillas *et al.*, 1978; Dumont *et al.*, 1975; Brown *et al.*, 1975; Portlock & Rosenberg 1977).

In the study of haematological findings at diagnosis in 140 cases by Bloomfield *et al.* (1977), the abnormalities in the blood did not correlate with survival in the absence of bone marrow involvement. Against this, the present study indicates that both high or low as opposed to normal neutrophil counts, low lymphocyte count, low platelet count and low haemoglobin are all associated with poor prognosis. With the exception of lymphocyte counts, and high neutrophil values, these associations remained apparent after adjustment for histopathology and symptoms and they would appear to provide useful secondary prognostic information. Our rate of detection of disease in the marrow in low grade lymphoma is somewhat lower than that of othet workers. This may have been due to the particle sectioning technique missing paratrabecular involvement, especially in follicle centre cell lymphomas. We have noticed this pattern in more recent studies where trephine biopsy technique has been used. However, the combination of multiple site aspiration and histological processing of particles in high grade lymphomas has produced a rate of detection comparable with results of other workers; (Dick *et al*., 1974; Rosenberg *et al*., 1975). Thus it is felt that our failure to find a correlation between bone marrow involvement and prognosis in patients with Stage II–IV high grade lymphoma, is unlikely to be due to failure to detect disease.

It may be postulated that the presence of systemic symptoms and disturbances of the acute phase proteins is related to prognosis by way of bulk of disease. Although disease bulk is very difficult to assess, an attempt was made to divide patients into those who had bulky and non-bulky disease and this showed a slight correlation with a presence of symptoms. However, when symptomatic stage was considered, the presence or absence of bulk disease was of only marginal value. It must be concluded that it is easier to assess symptoms rather than extent of bulk disease and that the exercise is more valuable. The effect upon prognosis of the elevation of C-reactive protein in asymptomatic patients is important and suggests that it might be wise to group these patients with those who have systemic symptoms.

This analysis indicates that it is reasonable to divide patients according to grade of malignancy and the presence or absence of symptoms in the first instance and that treatment should be based upon this subdivision providing that the stage of disease is beyond stage I. Within the four strata produced by this analysis it seems reasonable to treat the low grade asymptomatic patients purely for local problems following the principles of Portlock & Rosenberg (1977). Patients with low grade lymphoma and B symptoms will require active intervention. However, long-term results in this group with both combination and single agent chemotherapy have been disappointing (Portlock *et al.*, 1976; McKelvy & Moon, 1977; Lister *et al.*, 1978). Patients with disseminated high grade lymphoma all require aggressive treatment as their disease is potentially curable (DeVita *et al.*, 1975; Berd *et al.*, 1975; McKelvy & Moon, 1977). For patients with lymphoblastic lymphoma a completely different approach to chemotherapy is apparently required.

We are grateful to the Leukaemia Research Fund for their support of this study. A number of histopathologists kindly gave helpful advice on cases with difficult histology. We are particularly grateful to Dr E.L. Jones of the Department of Pathology, University of Birmingham in this respect.

### References

BERD, D., CORNOG, J., DECONTI, R.C., LEVITT, M. & BERTINO, J.R. (1975). Long-term remission in diffuse histiocytic lymphoma treated with combination sequential chemotherapy. *Cancer*, **35**, 1050.

BITRAN, J.D., KINZIE, J., SWEET, D.L. & 6 others. (1977). Survival of patients with localised histiocytic lymphoma. *Cancer*, **39**, 342.

BLOOMFIELD, C.D., GOLDMAN, A., DICK, R., BRUNNING, R.D. & KENNEDY, B.J. (1974). Multivariate analysis of prognostic factors in the non-Hodgkin's malignant lymphomas. *Cancer*, **33**, 870.

BLOOMFIELD, C.D., KERSEY, J.H., BRUNNING, R.D. & GAJL-PECZALSKA, K.J. (1976*a*). Prognostic significance of lymphocyte surface markers in adult non-Hodgkin's malignant lymphoma. *Cancer*, **ii**, 1330.

BLOOMFIELD, C.D., MCKENNA, R.W. & BRUNNING, R.D. (1976*b*). Significance of haematological parameters in non-Hodgkin's malignant lymphomas. *Br. J. Haematol.*, **32**, 41.

BLOOMFIELD, C.D., KERSEY, J.H., BRUNNING, R.D. & GAJL-PECZALSKA, K.J. (1977). Pognostic significance of lymphocyte surface markers and histology in adult non-Hodgkin's lymphomas. *Cancer Treat. Rep.*, **61**, 963.

BROWN, T.C., PETERS, M.V., BERGSAGEL, D.E. & REID, J. (1975). A retrospective analysis of the clinical results in relation to Rappaport histological classification. *Br. J. Cancer*, **31**, 174.

CABANILLAS, F., BAIKE, J.S., SMITH, T.L., MOON, T.E., BUTLER, J.J. & RODRIGUEZ, V. (1978). Factor predictive for response and survival in adults with advanced non-Hodgkin's lymphoma. *Arch. Int. Med.*, **138**, 413.

CARBONE, P.P., KAPLAN, H.S., MUSSHOFF, K., SMITHERS, D.W. & TUBIANA, M. (1971). Report of the committee on Hodgkin's Disease staging classification. *Cancer Res.*, **31**, 1860.

CHEN, M.G., PROSNITZ, L.R., GONZALEZ-SERVA, A. & FISCHER, D.B. (1979). Results of radiotherapy in control of stage I and II non-Hodgkin's lymphoma. *Cancer*, **43**, 1245.

DEVITA, V.T. Jr., CANELLOS, G.P., CHABNER, B., SCHEIN, P., HUBBARD, S.P. & YOUNG, R.C. (1975). Advanced diffuse histiocytic lymphoma, a potentially curable disease. Results with combination chemotherapy. *Lancet*, **i**, 248.

DICK, F., BLOOMFIELD, C.D. & BRUNNING, R.D. (1974). Incidence, cytology, and histopathology of non-Hodgkin's lymphomas in the bone marrow. *Cancer,* **33,** 1382.

DUMONT, J., DUFFILLOT, C., FLANDRIN, G., CHELLOAL, N., TRISFAUT, M. & BERNARD, J. (1975). Non-Hodgkin's lymphomata: clinical and immunological data in relation to histology. *Br. J. Cancer,* **31,** 187.

FISHER, R.I., DEVITA, V.T. Jr., JOHNSON, B.L., SIMON, R. & YOUNG, R.C. (1979). Prognostic factors for advanced diffuse histiocytic lymphoma following treatment with combination chemotherapy. *Am. J. Med.,* **63,** 177.

GERARD-MARCHANT, R., HAMLIN, J., LENNERT, K., RILKE, F., STANSFELD, A.G. & VAN UNNIK, J.A.M. (1974). Classification of non-Hodgkin's lymphomas. *Lancet,* **ii,** 406.

GLICK, J.H., MCFADDEN, E., COSTELLO, W., EZELINILI, E., BERNARD, C. & BENNETT, J. (1982). Nodular histiocytic lymphoma: factors influencing prognosis and implications for aggressive chemotherapy. *Cancer,* **49,** 840.

JONES, S.E., FUKS, Z., BULL, M., KADIN, M.E., DORFMAN, R.F., KAPLAN, H.S., ROSENBERG, S.A. & KIM, H. (1973). Non-Hodgkin's lymphomas. IV. Clinicopathologic correlation in 405 cases. *Cancer,* **31,** 806.

LENNERT, K. (1978). Handbuch des Speziellen Pathologischen Anatomie und Histologie. Berlin: Springer-Verlag.

LEVITT, S.H., BLOOMFIELD, C.D., FRIZZERA, G. & LEE, C.K.K. (1980). Curative radiotherapy for localised diffuse histiocytic lymphoma. *Cancer Treat. Rep.,* **64,** 175.

LISTER, T.A., CULLEN, M.H., BEARD, M.E.J. & 7 others. (1978). Comparison of combined and single agent chemotherapy in non-Hodgkin's lymphoma of favourable histological type. *Blood,* **54,** 1249.

LUKES, R.J. & COLLINS, R. (1975). New approaches to the classification of lymphomata. *Br. J. Cancer,* **31,** (Suppl. 2), 1.

MANCINI, G., CARBONARA, A.O. & HERLMANS, J.F. (1965). Immunochemical quantitation of antigens by single radial immunodiffusion. *Immunochemistry,* **2,** 235.

MCKELVEY, E.M. & MOON, T.E. (1977). Curability of non-Hodgkin's lymphoma. *Cancer Treat. Rep.,* **61,** 1185.

MEUSERS, P. (Kiel lymphoma study group). (1979). Heterogeneity of diffuse "histiocytic" lymphoma according to the Kiel classification. *N. Eng. J. Med.,* **301,** 384.

MEUSERS, P., KONIG, E. & BRETTINGER, G. (1980). Why not adhere to the original Kiel classification? (Letter) *Lancet,* **ii,** 1194.

PETO, R., PIKE, D., ARMITAGE, P. & 7 others. (1976). Design and analysis of randomised clinical trials requiring prolonged observation of each patient. I. Introduction and design. *Br. J. Cancer,* **34,** 505.

PETO, R., PIKE, M.C., ARMITAGE, P. & 7 others (1977). Design and analysis of randomized clinical trials which require prolonged observation of each patient. II Analysis and example. *Br. J. Cancer,* **35,** 1.

PORTLOCK, C.S., ROSENBERG, S.A., GLATSTEIN, E. & KAPLAN, H.S. (1976). Treatment of advanced non-Hodgkin's lymphomas with favourable histologies: Preliminary results of a prospective trial. *Blood,* **47,** 747.

PORTLOCK, C.S. & ROSENBERG, S.A. (1977). Chemotherapy of the non-Hodgkin's lymphomas: the Stanford experience. *Cancer Treat. Rep.,* **61,** 1049.

ROSENBERG, S.A., DORFMAN, R.F. & KAPLAN, M.S. (1975). The value of sequential bone marrow biopsy and splenectomy in a series of 127 consecutive untreated patients with non-Hodgkin's lymphoma. *Br. J. Cancer,* **2,** 168.

RUDDERS, R.A., KADDIS, M., DELELLIS, R.A. & CASEY, H. Jr. (1979). Nodular non-Hodgkin's lymphoma (NHL). Factors influencing prognosis and indications for aggressive treatment. *Cancer,* **43,** 1643.

WEINGRAD, D.S., DECOSSE, J.J., SHERLOCK, P., STRAUS, M.D., LIEBERMAN, P.H. & FLIPPA, D.A. (1982). Primary gastro-intestinal lymphoma. *Cancer,* **49,** 1258.